# Supplementary material for: Dietary Inflammatory Index and Dietary Diversity Score Associated with Sarcopenia and Its Components: Findings from a Nationwide Cross-Sectional Study
Source: Nutrients. 2024 Apr 2;16(7):1038. doi: 10.3390/nu16071038 (PMC11013103; doi:10.3390/nu16071038)
Supplement: Supplementary file 1 [file nutrients-16-01038-s001.zip › nutrients-2933251-supplementary.pdf]

Supplementary Materials

Associations of dietary diversity and inflammatory diet with sarcopenia and its components: findings from a nationwide cross-sectional study

Guzhengyue Zheng, Hui Xia, Zhihan Lai, Hui Shi, Junguo Zhang, Chongjian Wang, Fei Tian, Hualiang Lin

Contents

The definition of sarcopenia's components 3

Assessment of dietary diversity score (DDS) based on food frequency questionnaires (FFQ) in UK Biobank 4

Assessment of alcohol consumption and physical activity 5

Table S1 Distribution of baseline characteristics of participants before and after excluding from analyses 7

Table S2 The Data-Field for the key variables in this analysis 9

Table S3 Food items and mixed dishes involved in calculating DDS using 24-h dietary recalls information in UK Biobank 10

Table S4 Sensitivity analysis of associations of E-DII and DDS with sarcopenia while redefining sarcopenia following the EWGSOP1's recommendations 13

Table S5 Sensitivity analysis of associations of E-DII with sarcopenia and its components while excluding 6,336 participants with extreme BMI (*n* = 149,333) 15

Table S6 Sensitivity analysis of associations of E-DII with sarcopenia and its components while restricting the analysis to individuals who participated in at least two 24-h dietary surveys (*n* = 96,133) 18

Table S7 Sensitivity analysis of associations of E-DII with sarcopenia and its components when all missing covariates were imputed using multiple imputations (*n* = 202,708) 21

Table S8 Sensitivity analysis of ORs (95% CIs) of DDS and sarcopenia and its components while using FFQ information to calculate DDS in UK Biobank (*n* = 304,723) 24

Table S9 Sensitivity analysis of associations of DDS with sarcopenia and its components while excluding 6336 participants with extreme BMI (*n* = 149,333) 27

Table S10 Sensitivity analysis of associations of DDS with sarcopenia and its components while restricting the analysis to individuals who participated in at least two 24-h dietary surveys (*n* = 96,133) 30

Table S11 Sensitivity analysis of associations of DDS with sarcopenia and its components when all missing covariates were imputed using multiple imputations (*n* = 202,708) 33

Table S12 Sensitivity analysis of combined associations of E-DII and DDS with sarcopenia while redefining sarcopenia following the EWGSOP1's recommendations 36

Table S13 Sensitivity analysis of combined associations of E-DII and DDS with sarcopenia and its components while excluding participants with extreme BMI 38

Table S14 Sensitivity analysis of combined associations of E-DII and DDS with sarcopenia and its components while restricting the analysis to individuals who participated in at least two 24-h dietary surveys (*n* = 96,133) 41

Table S15 Sensitivity analysis of combined associations of E-DII and DDS with sarcopenia and its components when all missing covariates were imputed using multiple imputations (*n* = 202,708) 44

Table S16 Subgroup analyses of associations of E-DII with sarcopenia and its components 47

Table S17 Subgroup analyses of associations of DDS with sarcopenia and its components .. 49

Figure S1 Flow chart for UK Biobank using 24-h dietary assessment data 51

Figure S2 Timeline of five rounds of 24-hour dietary recall surveys in UK Biobank 52

Figure S3 Flow chart for UK Biobank using Food Frequency Questionnaire data 53

Reference 54

The definition of sarcopenia's components

An equation calculated RALM: RALM (kg/m^2) = [(0.958 × [appendicular fat-free mass (kg)] – (0.166 × G) -0.308)]/height^2 [1]. In this formula, appendicular fat-free mass was measured using a Tanita BC-418MA body composition bioimpedance analyzer, with a G value of 0 in the case of females and 1 in the case of males, and height referred to standing height. Following the EWGSOP2 cut-off points, low RALM was defined as 5.5 kg/m^2 for females and less than 7.0 kg/m^2 for males.

Jamar hydraulic hand dynamometers were used as hand grip strength measurements. Both the left and right hands were measured (in kg), and the larger grip strength value was used to define low hand grip strength. The thresholds of low hand grip strength for different genders are as follows: <27 kg among males and <16 kg among females. Participants who could not complete grip strength measurement (mainly because of disease reasons: stroke, arthritis, or other health issues) in either hand were also considered to have low hand grip strength.

In the EWGSOP2, low physical performance was defined by a walking pace <0.8 meters per second. In the UK Biobank, walking pace was self-reported and classified into slow (<3 miles per hour), steady/average (3 to 4 miles per hour), and fast (>4 miles per hour). Therefore, we used a walking pace of <3 miles per hour as the criterion for low physical performance (equivalent to <1.3 meters per second) [2].

Assessment of dietary diversity score (DDS) based on food frequency questionnaires (FFQ) in UK Biobank

In UK Biobank, 502,461 participants completed a brief touchscreen food frequency questionnaire (FFQ) with 33 dietary information about beverages, main foods, and food consumed over the previous year at baseline. Among the 502,461 participants, those meeting the following criteria were excluded in this study: whose dietary information was missing or unbelievable, who had withdrawn consent for linkage, who had missing data on key covariates, or who had been diagnosed or taken diabetes related medicines or self-reported diabetes. The remaining 304,723 participants were included in the analysis.

DDS was assessed based on FFQ, related to15 major food groups: processed meat, beef, mutton, pork, poultry, oily fish, non-oily fish, cheese, milk, cereal, bread, cooked vegetables, raw vegetables, fresh fruits, and dried fruits. Participants were asked how often processed meat, beef, mutton, pork, poultry, oily fish, non-oily fish, and cheese were consumed with eight choices: “never eaten”, “<1 time a week”, “1 time a week”, “2-4 times a week”, “5-6 times a week”, “≥1 time daily”, “do not know”, and “prefer not to answer”. For cereal, bread, cooked vegetables, raw vegetables, fresh fruit, and dried fruit, participants answered the integer number of bowls/slices/heaped tablespoons/pieces of each item. The participants also had three other options to select: “less than one”, “do not know”, or “prefer not to answer”. Responses of “do not know”

or “prefer not to answer” for a specific dietary item were converted into missing values. Consumption of any food group without considering a minimum amount was categorized as one point. We consider that a person's normal diet is impossible to eat nothing for a day, the DDS ranges from 1 – 15. A higher DDS score reflects a richer diet and vice versa. We divided DDS score into three categories: low DDS (ranges 1 – 5), medium DDS (ranges 6 – 10), and high DDS (ranges 11 – 15) and the reference group was set as the participants with the low DDS category.

Assessment of alcohol consumption and physical activity

Alcohol consumption: In the current analysis, alcohol intake (g/week) was calculated by the quantity of each type of drink (red wine, white wine, beer/cider, fortified wine, and spirits) multiplied by its standard drink size and reference alcohol content [1 drink-equivalent described as containing 8 g of pure alcohol; 125 mL wine (red or white) = 1.6 unit-equivalents, 1 pint of beer (586 mL) = 2.6 unit-equivalents, 25mL spirits =1 unit-equivalent, 62.5 mL fortified wine = 1unit-equivalent] [3].

Physical activity: Physical activity (PA) was ascertained with the International Physical Activity Questionnaire (IPAQ) [4], which includes six questions about duration and frequency of walking, moderate-intensity and vigorous-intensity exercise undergone in the last 4 weeks [4]. Each intensity was assigned a corresponding metabolic equivalent of task (METs): 3.3 for walking, 4.0 for moderate PA, and 8.0 for vigorous PA. MET is an objective measurement of the ratio of energy expenditure rate

to an individual's mass (1 MET = 1kilocalorie per hour per kilogram of bodyweight) [5]. We then quantified PA of each participant by calculating hours of MET each week (MET-hour/week) based on the reported intensity, duration, and frequency of PA in 1 week [6].

Table S1 Distribution of baseline characteristics of participants before and after excluding from analyses [a]

Characteristics	Before excluding (n = 211,025)	After excluding (n = 155,669)
Age, years, mean (SD)	56.1 (8.0)	55.9 (7.9)
Sex, female	115,867 (55.2)	80,014 (51.4)
Race, nonwhite	9597 (4.6)	6227 (4.0)
Residence, rural	33,499 (16.0)	25,063 (16.1)
Household income		
High	59,483 (28.4)	51,215 (32.9)
Medium	99,275 (47.3)	81,726 (52.5)
Low	29,165 (13.9)	22,572 (14.5)
Smoking status		
Never	118,559 (56.5)	87,486 (56.2)
Previous	74,242 (35.4)	56,197 (36.1)
Current	16,425 (7.8)	11,987 (7.7)
Alcohol consumption, g/week, mean (SD)	110.5 (97.6)	115.6 (99.9)
PA, MET (hours/week), mean (SD)	41.6 (37.8)	40.9 (40.2)
BMI (kg/m2), mean (SD)	26.1 (4.7)	26.9 (4.5)
Energy intake, kcal/day,	2119.3 (739.5)	2089.2 (617.4)

mean (SD)		
Dietary supplement	100,308 (47.8)	73,456 (47.2)
Diabetes	9074 (4.3)	6345 (4.1)
CVD	8675 (4.1)	6694 (4.3)
Cancer	18,311 (8.7)	13,232 (8.5)
Hypertension	90,380 (43.1)	39,540 (25.4)
Hyperlipidemia	34,188 (16.3)	19,147 (12.3)
Low muscle strength	8543 (4.0)	5760 (3.7)
Low muscle mass	12,377 (5.9)	8717 (5.6)
Low physical performance	11,135 (5.3)	7316 (4.7)

[a] Values are numbers (percentages) unless stated otherwise. Abbreviations: BMI, body mass index; CVD, cardiovascular disease; MET, metabolic equivalent; PA, physical activity; SD, standard deviation.

Table S2 The Data-Field for the key variables in this analysis

Variables	Data-Field
Age	21022
Sex	31
Race	21000
Residential area	20118
Household income	738
Smoking status	20116
Physical activity	22032
Alcohol consumption	100022
BMI	21001
Energy intake	26002
Dietary supplement	104670, 20084
Diabetes	130708, 130709, 30750, 2976, 20003
CVD	6150, 3894, 4056, 131368, 42008, 42006
Cancer	2453, 20001-0.0~0.5, 40006-0.0~16.0
Hypertension	93-0.0~0.1, 94-0.0~0.1, 6177-0.0~0.2
High cholesterol	20002-0.0~0.33, 6153-0.0~0.3, 6177-0.0~0.3
Low muscle strength	46, 47
Low muscle mass	23113, 23117, 23121, 23125, 50
Low physical performance	924

Abbreviations: BMI, body mass index; CVD, cardiovascular disease.

Table S3 Food items and mixed dishes involved in calculating DDS using 24-h dietary recalls information in UK Biobank

Major food groups	Subgroups	Representative food items and mixed dishes on the 24-h dietary recalls
**Grain products**	Whole grains	Porridge, oat crunch, bran cereal, whole-wheat cereal, whole meal sliced bread, whole meal baguette, whole meal bap, whole meal bread roll, crispbread, oat cakes, whole meal pasta, brown rice, couscous, other whole grains
	Non-whole grains	Muesli, plain cereal, dried fruit cereal, plain sliced bread, plain baguette, plain bap, plain bread roll, naan bread, garlic bread, other non-whole grain bread, white pasta, white rice, sushi, snack pot, pizza, pancakes, scotch pancakes, Danish pastry, cake, other non-whole grains
**Vegetables**	Dark green leafy	Spinach, broccoli, watercress, beetroot
	Vitamin A-rich	Carrots, sweet peppers

	Starchy tubers	Potatoes, boiled potatoes, mashed potatoes, sweet potatoes, butternut squash, sweet corn
	Other	Vegetable pieces, (mixed) salad, cabbage, cauliflower, celery, courgetti, cucumber, garlic, leeks, lettuce, mushrooms, onions, parsnip, fresh tomatoes, tinned tomatoes, turnip, other vegetables
**Fruits**	Citrus	Grape, oranges, satsuma
	Vitamin A-rich	Melon, peach
	other	Mixed fruits, bananas, berry, cherry, grape, mango, pear, pineapple, plum, olives, avocados, other fruits
**Meat and protein alternatives**	Red meat	Beef, pork, mutton, burger, bacon, ham, corned beef, sausages, lasagna, meat soup, quiche, savory pies
	Poultry	Deep fried chicken, chicken, other poultry
	Fish and seafood	Tinned tuna, oily fish, breaded fish, battered fish, white fish, prawns, lobster, shellfish, other fish

	Organ meat	Liver, kidney
	Eggs	Whole eggs, scrambled eggs, eggs in sandwiches, scotch eggs, other eggs
	Legumes, nuts, and seeds	Soya dessert, salted peanuts, unsalted peanuts, salted nuts, unsalted nuts, seeds, tofu, baked beans, broad beans, green beans, other beans, peas
**Dairy products**	Milk	Milk (fortified, goat's, sheep's, etc.), added milk to coffee, added milk to tea, ice cream, lasagna, quiche
	Yoghurt	Yogurt smoothie, flavored milk, ice cream, yogurt (whole milk, low fat)
	Cheese	Cheese, low fat (spreadable) cheese, hard cheese, soft cheese, blue cheese, spreadable cheese, cottage cheese, feta, mozzarella, goat cheese, other cheese

Abbreviation: DDS, dietary diversity score.

Table S4 Sensitivity analysis of associations of E-DII and DDS with sarcopenia while redefining sarcopenia following the EWGSOP1's recommendations [a]

	Total n/events	Model 1 (ORs, 95% CIs)	Model 2 (ORs, 95% CIs)	Model 3 (ORs, 95% CIs)
E-DII [d]				
Tertile 3 [b]	51,890/4436	1.00 (Ref)	1.00 (Ref)	1.00 (Ref)
Tertile 2 [b]	51,889/4731	0.68 (0.55, 0.85)	0.72 (0.69, 0.77)	0.73 (0.63, 0.97)
Tertile 1 [b]	51,890/5036	0.70 (0.58, 0.85)	0.70 (0.66, 0.74)	0.75 (0.62, 0.90)
Continuous (per SD reduction)	155,669/14,203	0.92 (0.90, 0.93)	0.94 (0.93, 0.96)	0.96 (0.94, 0.98)
DDS [e]				
Low [c]	18,838/1634	1.00 (Ref)	1.00 (Ref)	1.00 (Ref)
Medium [c]	95,428/8543	0.74 (0.58, 0.95)	0.74 (0.70, 0.80)	0.85 (0.68, 0.98)
High [c]	41,403/4026	0.66 (0.50, 0.87)	0.73 (0.68, 0.79)	0.78 (0.61, 0.89)
Continuous (per SD increment)	155,669/14,203	0.94 (0.92, 0.95)	0.95 (0.94, 0.97)	0.97 (0.96, 0.99)

[a] We defined sarcopenia as having low muscle mass and either low muscle strength or low physical performance following the EWGSOP1's recommendations.

[b] Tertile 1 of E-DII ranged from -5.45 to -0.49, tertile 2 of E-DII ranged from -0.50 to 1.12, and tertile 3 of E-DII ranged from 1.13 to 4.65.

[c] DDS categories [low (1-6), medium (7-12), and high (13-18)] were defined according to practical implications for public health.

[d] ORs (95% CIs) of E-DII with sarcopenia were examined using Logistic regression models; model 1 was adjusted for age and sex; model 2 was further adjusted for race, household income, residence, smoking status, alcohol consumption, physical activity, and dietary supplement; model 3 additionally included BMI, diabetes, CVD, cancer, hypertension, and hyperlipidemia.

[e] ORs (95% CIs) of DDS with sarcopenia were examined using Logistic regression models; model 1 was adjusted for age and sex; model 2 was further adjusted for race, household income, residence, smoking status, alcohol consumption, physical activity, dietary supplement, and total calorie intake from diet; model 3 additionally included BMI, diabetes, CVD, cancer, hypertension, and hyperlipidemia.

Abbreviations: BMI, body mass index; CIs: confidence intervals; CVD, cardiovascular disease; DDS, dietary diversity score; E-DII, dietary inflammatory index; ORs: odds ratios; Ref, reference; SD, standard deviation.

Table S5 Sensitivity analysis of associations of E-DII with sarcopenia and its components while excluding 6,336 participants with extreme BMI (*n* = 149,333) [a]

Models	E-DII (ORs, 95% CIs)			
	**Tertile 1 (-5.45, -0.50)**	**Tertile 2 (-0.51, 1.13)**	**Tertile 3 (1.14, 4.65)**	**Continuous (per SD reduction)**
No. of participants	49,901	49,953	49,479	149,333
Sarcopenia				
Cases	156	146	194	496
Model 1 [b]	0.71 (0.56, 0.89)	0.71 (0.57, 0.88)	1.00 (Ref)	0.85 (0.78, 0.93)
Model 2 [b]	0.71 (0.53, 0.93)	0.75 (0.59, 0.94)	1.00 (Ref)	0.83 (0.75, 0.91)
Model 3 [b]	0.76 (0.56, 0.99)	0.80 (0.64, 1.01)	1.00 (Ref)	0.89 (0.81, 0.97)
Low muscle strength				
Cases	1690	1766	1987	5443

Model 1 [c]	0.78 (0.73, 0.84)	0.84 (0.78, 0.89)	1.00 (Ref)	0.89 (0.87, 0.92)
Model 2 [c]	0.83 (0.77, 0.90)	0.88 (0.82, 0.94)	1.00 (Ref)	0.92 (0.89, 0.94)
Model 3 [c]	0.82 (0.76, 0.89)	0.88 (0.82, 0.94)	1.00 (Ref)	0.92 (0.89, 0.94)
Low muscle mass				
Cases	2793	3022	3112	8927
Model 1 [c]	0.80 (0.74, 0.87)	0.93 (0.87, 1.00)	1.00 (Ref)	0.86 (0.83, 0.88)
Model 2 [c]	0.82 (0.77, 0.87)	0.88 (0.83, 0.94)	1.00 (Ref)	0.87 (0.85, 0.90)
Model 3 [c]	0.89 (0.83, 0.96)	0.92 (0.87, 0.98)	1.00 (Ref)	0.88 (0.86, 0.91)
Low physical performance				
Cases	1625	1682	2379	5686
Model 1 [c]	0.63 (0.59, 0.67)	0.66 (0.62, 0.71)	1.00 (Ref)	0.79 (0.77, 0.82)
Model 2 [c]	0.73 (0.68, 0.79)	0.74 (0.69, 0.79)	1.00 (Ref)	0.87 (0.85, 0.90)
Model 3 [c]	0.75 (0.70, 0.81)	0.76 (0.71, 0.82)	1.00 (Ref)	0.88 (0.86, 0.91)

[a] Extreme BMI was defined as low (<14 kg/m^2) or high (≥36 kg/m^2) BMI.

[b] ORs (95% CIs) of E-DII with sarcopenia were examined using Logistic regression models; model 1 was adjusted for age and sex; model 2 was further adjusted for race, household income, residence, smoking status, alcohol consumption, physical activity, and dietary supplement; model 3 additionally included BMI, diabetes, CVD, cancer, hypertension, and hyperlipidemia.

[c] ORs (95% CIs) of E-DII with sarcopenia's components were examined using Logistic regression models; model 1 was adjusted for age and sex; model 2 was further adjusted for race, household income, residence, smoking status, alcohol consumption, physical activity, dietary supplement and three components (low muscle strength, low muscle mass, and low physical performance) were mutually adjusted; model 3 additionally included BMI, diabetes, CVD, cancer, hypertension, and hyperlipidemia.

Abbreviations: BMI, body mass index; CIs: confidence intervals; CVD, cardiovascular disease; E-DII, dietary inflammatory index; ORs: odds ratios; Ref, reference; SD, standard deviation.

Table S6 Sensitivity analysis of associations of E-DII with sarcopenia and its components while restricting the analysis to individuals who participated in at least two 24-h dietary surveys (n = 96,133)

Models	E-DII (ORs, 95% CIs)			
	**Tertile 1 (-5.21, -0.50)**	**Tertile 2 (-0.51, 1.13)**	**Tertile 3 (1.14, 4.52)**	**Continuous (per SD reduction)**
No. of participants	32,599	34,537	28,997	96,133
Sarcopenia				
Cases	93	95	115	303
Model 1 [a]	0.63 (0.47, 0.84)	0.68 (0.51, 0.90)	1.00 (Ref)	0.77 (0.69, 0.87)
Model 2 [a]	0.66 (0.49, 0.88)	0.70 (0.54, 0.91)	1.00 (Ref)	0.80 (0.71, 0.90)
Model 3 [a]	0.70 (0.52, 0.94)	0.74 (0.57, 0.96)	1.00 (Ref)	0.83 (0.74, 0.93)
Low muscle strength				
Cases	1027	1186	1075	3288

Model 1 [b]	0.79 (0.73, 0.87)	0.88 (0.81, 0.96)	1.00 (Ref)	0.90 (0.87, 0.93)
Model 2 [b]	0.83 (0.76, 0.91)	0.92 (0.84, 1.00)	1.00 (Ref)	0.92 (0.89, 0.95)
Model 3 [b]	0.83 (0.76, 0.91)	0.92 (0.84, 1.00)	1.00 (Ref)	0.92 (0.89, 0.95)
Low muscle mass				
Cases	1876	2139	1940	5955
Model 1 [b]	0.65 (0.60, 0.72)	0.82 (0.75, 0.90)	1.00 (Ref)	0.79 (0.76, 0.82)
Model 2 [b]	0.90 (0.83, 0.96)	0.90 (0.84, 0.96)	1.00 (Ref)	0.92 (0.89, 0.94)
Model 3 [b]	0.90 (0.84, 0.97)	0.90 (0.84, 0.97)	1.00 (Ref)	0.92 (0.90, 0.95)
Low physical performance				
Cases	1167	1268	1558	3993
Model 1 [b]	0.63 (0.58, 0.68)	0.65 (0.60, 0.70)	1.00 (Ref)	0.80 (0.77, 0.83)
Model 2 [b]	0.73 (0.67, 0.80)	0.77 (0.63, 0.75)	1.00 (Ref)	0.88 (0.85, 0.91)
Model 3 [b]	0.76 (0.70, 0.83)	0.78 (0.72, 0.84)	1.00 (Ref)	0.88 (0.85, 0.92)

[a] ORs (95% CIs) of E-DII with sarcopenia were examined using Logistic regression models; model 1 was adjusted for age and sex; model 2 was further adjusted for race, household income, residence, smoking status, alcohol consumption, physical activity, and dietary supplement; model 3 additionally included BMI, diabetes, CVD, cancer, hypertension, and hyperlipidemia.

[b] ORs (95% CIs) of E-DII with sarcopenia's components were examined using Logistic regression models; model 1 was adjusted for age and sex; model 2 was further adjusted for race, household income, residence, smoking status, alcohol consumption, physical activity, dietary supplement and three components (low muscle strength, low muscle mass, and low physical performance) were mutually adjusted; model 3 additionally included BMI, diabetes, CVD, cancer, hypertension, and hyperlipidemia.

Abbreviations: BMI, body mass index; CIs: confidence intervals; CVD, cardiovascular disease; E-DII, dietary inflammatory index; ORs: odds ratios; Ref, reference; SD, standard deviation.

Table S7 Sensitivity analysis of associations of E-DII with sarcopenia and its components when all missing covariates were imputed using multiple imputations (n = 202,708)

Models	E-DII (ORs, 95% Cls)			
	**Tertile 1 (-5.44, -0.46)**	**Tertile 2 (-0.47, 1.17)**	**Tertile 3 (1.18, 4.72)**	**Continuous (per SD reduction)**
No. of participants	67,569	67,569	67,570	202,708
Sarcopenia				
Cases	229	223	278	730
Model 1 [a]	0.75 (0.62, 0.90)	0.77 (0.65, 0.92)	1.00 (Ref)	0.85 (0.78, 0.91)
Model 2 [a]	0.78 (0.65, 0.94)	0.81 (0.68, 0.97)	1.00 (Ref)	0.86 (0.80, 0.93)
Model 3 [a]	0.80 (0.67, 0.97)	0.84 (0.70, 1.00)	1.00 (Ref)	0.89 (0.82, 0.96)
Low muscle strength				

Cases	2514	2678	3074	8266
Model 1 [b]	0.77 (0.73, 0.81)	0.83 (0.79, 0.88)	1.00 (Ref)	0.88 (0.86, 0.90)
Model 2 [b]	0.79 (0.75, 0.84)	0.87 (0.82, 0.92)	1.00 (Ref)	0.90 (0.88, 0.92)
Model 3 [b]	0.83 (0.78, 0.87)	0.88 (0.84, 0.93)	1.00 (Ref)	0.91 (0.89, 0.93)
Low muscle mass				
Cases	3833	4082	4180	12,095
Model 1 [b]	0.90 (0.85, 0.94)	0.90 (0.85, 0.94)	1.00 (Ref)	0.94 (0.92, 0.96)
Model 2 [b]	0.90 (0.85, 0.95)	0.90 (0.86, 0.95)	1.00 (Ref)	0.94 (0.92, 0.96)
Model 3 [b]	0.91 (0.87, 0.96)	0.91 (0.87, 0.96)	1.00 (Ref)	0.95 (0.93, 0.97)
Low physical performance				
Cases	3009	3126	4522	10,657
Model 1 [b]	0.62 (0.60, 0.66)	0.66 (0.63, 0.69)	1.00 (Ref)	0.79 (0.78, 0.81)
Model 2 [b]	0.65 (0.62, 0.68)	0.69 (0.66, 0.73)	1.00 (Ref)	0.81 (0.80, 0.83)

Model 3 [b]	0.73 (0.69, 0.77)	0.74 (0.70, 0.77)	1.00 (Ref)	0.87 (0.85, 0.88)

[a] ORs (95% CIs) of E-DII with sarcopenia were examined using Logistic regression models; model 1 was adjusted for age and sex; model 2 was further adjusted for race, household income, residence, smoking status, alcohol consumption, physical activity, and dietary supplement; model 3 additionally included BMI, diabetes, CVD, cancer, hypertension, and hyperlipidemia.

[b] ORs (95% CIs) of E-DII with sarcopenia's components were examined using Logistic regression models; model 1 was adjusted for age and sex; model 2 was further adjusted for race, household income, residence, smoking status, alcohol consumption, physical activity, dietary supplement and three components (low muscle strength, low muscle mass, and low physical performance) were mutually adjusted; model 3 additionally included BMI, diabetes, CVD, cancer, hypertension, and hyperlipidemia.

Abbreviations: BMI, body mass index; CIs: confidence intervals; CVD, cardiovascular disease; E-DII, dietary inflammatory index; ORs: odds ratios; Ref, reference; SD, standard deviation.

Table S8 Sensitivity analysis of ORs (95% CIs) of DDS and sarcopenia and its components while using FFQ information to calculate DDS in UK Biobank (n = 304,723)

	**DDS categories (ORs, 95% CIs)**			**Continuous (per SD increment)**
	**Low [a] (n = 30,523)**	**Medium [a] (n = 259,863)**	**High [a] (n = 14,337)**	
Sarcopenia				
Cases	174	825	43	1042
Model 1 [b]	1.00 (Ref)	0.49 (0.41, 0.57)	0.42 (0.30, 0.59)	0.76 (0.71, 0.80)
Model 2 [b]	1.00 (Ref)	0.60 (0.51, 0.71)	0.56 (0.40, 0.78)	0.80 (0.75, 0.85)
Model 3 [b]	1.00 (Ref)	0.89 (0.67, 0.99)	0.78 (0.65, 0.93)	0.82 (0.77, 0.87)
Low muscle strength				
Cases	1502	10,007	594	12,103
Model 1 [c]	1.00 (Ref)	0.69 (0.66, 0.74)	0.69 (0.63, 0.76)	0.88 (0.87, 0.90)

Model 2 [c]	1.00 (Ref)	0.88 (0.80, 0.97)	0.83 (0.78, 0.87)	0.92 (0.91, 0.94)
Model 3 [c]	1.00 (Ref)	0.87 (0.79, 0.97)	0.82 (0.78, 0.87)	0.94 (0.92, 0.96)
Low muscle				
Cases	2247	14,023	616	16,886
Model 1 [c]	1.00 (Ref)	0.72 (0.69, 0.76)	0.60 (0.55, 0.66)	0.88 (0.86, 0.89)
Model 2 [c]	1.00 (Ref)	0.74 (0.71, 0.78)	0.63 (0.57, 0.69)	0.88 (0.87, 0.89)
Model 3 [c]	1.00 (Ref)	1.00 (0.88, 1.12)	0.92 (0.86, 0.98)	0.89 (0.87, 0.90)
Low physical performance				
Cases	2502	13,696	758	16,956
Model 1 [c]	1.00 (Ref)	0.72 (0.69, 0.76)	0.60 (0.55, 0.66)	0.83 (0.82, 0.84)
Model 2 [c]	1.00 (Ref)	0.88 (0.81, 0.96)	0.80 (0.76, 0.84)	0.88(0.87, 0.90)
Model 3 [c]	1.00 (Ref)	0.76 (0.69, 0.83)	0.74 (0.70, 0.78)	0.95 (0.93, 0.96)

[a] DDS category (based on FFQ) [low (1-5), medium (6-10), and high (11-15)] were defined according to practical implications for public health.

[b] ORs (95% CIs) of DDS with sarcopenia were examined using Logistic regression models; model 1 was adjusted for age and sex; model 2 additionally included race, household income, residence, smoking status, alcohol consumption, physical activity, dietary supplement, and total calorie intake from diet; model 3 additionally included BMI, diabetes, CVD, cancer, hypertension, and hyperlipidemia.

[c] ORs (95% CIs) of DDS with sarcopenia's components were examined using Logistic regression models; model 1 was adjusted for age and sex; model 2 additionally included race, household income, residence, smoking status, alcohol consumption, physical activity, dietary supplement, total calorie intake from diet, and three components (low muscle strength, low muscle mass, and low physical performance) were mutually adjusted; model 3 additionally included BMI, diabetes, CVD, cancer, hypertension, and hyperlipidemia.

Abbreviations: BMI, body mass index; CIs: confidence intervals; CVD, cardiovascular disease; DDS, dietary diversity score; FFQ, Food Frequency Questionnaire; ORs: odds ratios; Ref, reference; SD, standard deviation.

Table S9 Sensitivity analysis of associations of DDS with sarcopenia and its components while excluding 6336 participants with extreme BMI (n = 149,333) [a]

Model	DDS, ORs (95% CIs)			Continuous (per SD increment)
	Low (1-6)	Medium (7-12)	High (13-18)	
No. of participants	17,772	9,1511	40,050	149,333
Sarcopenia				
Cases	57	308	131	496
Model 1 [b]	1.00 (Ref)	0.79 (0.57, 1.09)	0.67 (0.46, 0.93)	0.83 (0.75, 0.91)
Model 2 [b]	1.00 (Ref)	0.79 (0.59, 1.08)	0.65 (0.77, 0.92)	0.85 (0.77, 0.93)
Model 3 [b]	1.00 (Ref)	0.80 (0.56, 1.15)	0.72 (0.47, 0.94)	0.90 (0.82, 0.99)
Low muscle strength				
Cases	771	3291	1381	5443
Model 1 [c]	1.00 (Ref)	0.71 (0.65, 0.77)	0.61 (0.56, 0.67)	0.86 (0.84, 0.88)

Model 2 [c]	1.00 (Ref)	0.76 (0.70, 0.82)	0.69 (0.63, 0.76)	0.89 (0.87, 0.92)
Model 3 [c]	1.00 (Ref)	0.77 (0.71, 0.83)	0.70 (0.64, 0.77)	0.89 (0.87, 0.92)
Low muscle mass				
Cases	851	5367	2709	8927
Model 1 [c]	1.00 (Ref)	0.88 (0.80, 0.96)	0.77 (0.69, 0.87)	0.95 (0.93, 0.98)
Model 2 [c]	1.00 (Ref)	0.83 (0.75, 0.93)	0.76 (0.68, 0.86)	0.96 (0.93, 0.98)
Model 3 [c]	1.00 (Ref)	0.88 (0.80, 0.97)	0.79 (0.71, 0.89)	0.94 (0.91, 0.96)
Low physical performance				
Cases	1051	3441	1194	5686
Model 1 [c]	1.00 (Ref)	0.56 (0.52, 0.60)	0.41 (0.38, 0.45)	0.74 (0.72, 0.76)
Model 2 [c]	1.00 (Ref)	0.67 (0.63, 0.72)	0.55 (0.51, 0.60)	0.82 (0.80, 0.84)
Model 3 [c]	1.00 (Ref)	0.74 (0.69, 0.79)	0.63 (0.58, 0.68)	0.85 (0.83, 0.87)

[a] Extreme BMI was defined as low (<14 kg/m^2) or high (≥36 kg/m^2) BMI.

[b] ORs (95% CIs) of DDS with sarcopenia were examined using Logistic regression models; model 1 was adjusted for age and sex; model 2 additionally included race, household income, residence, smoking status, alcohol consumption, physical activity, dietary supplement, and total calorie intake from diet; model 3 additionally included BMI, diabetes, CVD, cancer, hypertension, and hyperlipidemia.

[c] ORs (95% CIs) of DDS with sarcopenia's components were examined using Logistic regression models; model 1 was adjusted for age and sex; model 2 additionally included race, household income, residence, smoking status, alcohol consumption, physical activity, dietary supplement, total calorie intake from diet, and three components (low muscle strength, low muscle mass, and low physical performance) were mutually adjusted; model 3 additionally included BMI, diabetes, CVD, cancer, hypertension, and hyperlipidemia.

Abbreviations: BMI, body mass index; CIs: confidence intervals; CVD, cardiovascular disease; DDS, dietary diversity score; ORs: odds ratios; Ref, reference; SD, standard deviation.

Table S10 Sensitivity analysis of associations of DDS with sarcopenia and its components while restricting the analysis to individuals who participated in at least two 24-h dietary surveys (n = 96,133)

**Model**	**DDS, ORs (95% Cls)**			**Continuous (per SD increment)**
	**Low (1-6)**	**Medium (7-12)**	**High (13-18)**	
No. of participants	1781	53,747	40,505	96,133
Sarcopenia				
Cases	16	163	124	303
Model 1 [a]	1.00 (Ref)	0.63 (0.30, 1.31)	0.49 (0.23, 0.95)	0.81 (0.72, 0.92)
Model 2 [a]	1.00 (Ref)	0.76 (0.36, 1.34)	0.63 (0.30, 0.92)	0.80 (0.70, 0.91)
Model 3 [a]	1.00 (Ref)	0.80 (0.36, 1.36)	0.67 (0.30, 0.94)	0.85 (0.75, 0.92)
Low muscle strength				
Cases	69	1813	1406	3288

Model 1 [b]	1.00 (Ref)	0.67 (0.53, 0.87)	0.61 (0.48, 0.78)	0.91 (0.88, 0.94)
Model 2 [b]	1.00 (Ref)	0.74 (0.58, 0.95)	0.70 (0.54, 0.90)	0.94 (0.90, 0.97)
Model 3 [b]	1.00 (Ref)	0.75 (0.59, 0.97)	0.71 (0.55, 0.92)	0.94 (0.91, 0.97)
Low muscle mass				
Cases	87	3212	2656	5955
Model 1 [b]	1.00 (Ref)	0.57 (0.40, 0.86)	0.53 (0.37, 0.80)	0.75 (0.71, 0.78)
Model 2 [b]	1.00 (Ref)	0.73 (0.56, 0.98)	0.69 (0.51, 0.92)	0.79 (0.76, 0.81)
Model 3 [b]	1.00 (Ref)	0.78 (0.57, 1.03)	0.73 (0.54, 0.99)	0.78 (0.76, 0.81)
Low physical performance				
Cases	145	2392	1456	3993
Model 1 [b]	1.00 (Ref)	0.44 (0.37, 0.53)	0.33 (0.27, 0.39)	0.77 (0.75, 0.80)
Model 2 [b]	1.00 (Ref)	0.56 (0.47, 0.67)	0.47 (0.39, 0.57)	0.85 (0.82, 0.88)
Model 3 [b]	1.00 (Ref)	0.63 (0.52, 0.77)	0.55 (0.45, 0.67)	0.88 (0.85, 0.91)

[a] ORs (95% CIs) of DDS with sarcopenia were examined using Logistic regression models; model 1 was adjusted for age and sex; model 2 additionally included race, household income, residence, smoking status, alcohol consumption, physical activity, dietary supplement, and total calorie intake from diet; model 3 additionally included BMI, diabetes, CVD, cancer, hypertension, and hyperlipidemia.

[b] ORs (95% CIs) of DDS with sarcopenia's components were examined using Logistic regression models; model 1 was adjusted for age and sex; model 2 additionally included race, household income, residence, smoking status, alcohol consumption, physical activity, dietary supplement, total calorie intake from diet, and three components (low muscle strength, low muscle mass, and low physical performance) were mutually adjusted; model 3 additionally included BMI, diabetes, CVD, cancer, hypertension, and hyperlipidemia.

Abbreviations: BMI, body mass index; CIs: confidence intervals; CVD, cardiovascular disease; DDS, dietary diversity score; ORs: odds ratios; Ref, reference; SD, standard deviation.

Table S11 Sensitivity analysis of associations of DDS with sarcopenia and its components when all missing covariates were imputed using multiple imputations (*n* = 202,708)

Model	DDS, ORs (95% Cls)			Continuous (per SD increment)
	Low (1-6)	Medium (7-12)	High (13-18)	
No. of participants	25,855	123,961	52,892	202,708
Sarcopenia				
Cases	90	449	191	730
Model 1 [a]	1.00 (Ref)	0.74 (0.57, 0.96)	0.64 (0.48, 0.86)	0.85 (0.79, 0.92)
Model 2 [a]	1.00 (Ref)	0.83 (0.64, 1.07)	0.67 (0.47, 0.92)	0.85 (0.78, 0.92)
Model 3 [a]	1.00 (Ref)	0.70 (0.50, 0.97)	0.67 (0.45, 0.95)	0.86 (0.79, 0.94)
Low muscle strength				
Cases	1278	5053	1935	8266
Model 1 [b]	1.00 (Ref)	0.69 (0.65, 0.74)	0.57 (0.53, 0.61)	0.83 (0.81, 0.85)

Model 2 [b]	1.00 (Ref)	0.75 (0.70, 0.80)	0.65 (0.60, 0.70)	0.87 (0.85, 0.89)
Model 3 [b]	1.00 (Ref)	0.77 (0.72, 0.82)	0.67 (0.62, 0.72)	0.87 (0.85, 0.89)
Low muscle mass				
Cases	1230	7283	3582	12,095
Model 1 [b]	1.00 (Ref)	0.88 (0.82, 0.95)	0.85 (0.78, 0.93)	0.96 (0.94, 0.98)
Model 2 [b]	1.00 (Ref)	0.89 (0.83, 0.96)	0.87 (0.79, 0.95)	0.96 (0.94, 0.98)
Model 3 [b]	1.00 (Ref)	0.92 (0.85, 0.99)	0.91 (0.83, 0.99)	0.97 (0.95, 0.99)
Low physical performance				
Cases	2076	6488	2093	10,657
Model 1 [b]	1.00 (Ref)	0.56 (0.53, 0.59)	0.39 (0.37, 0.42)	0.73 (0.71, 0.74)
Model 2 [b]	1.00 (Ref)	0.62 (0.59, 0.66)	0.47 (0.44, 0.50)	0.77 (0.75, 0.78)
Model 3 [b]	1.00 (Ref)	0.67 (0.63, 0.71)	0.53 (0.50, 0.57)	0.80 (0.79, 0.82)

[a] ORs (95% CIs) of DDS with sarcopenia were examined using Logistic regression models; model 1 was adjusted for age and sex; model 2

additionally included race, household income, residence, smoking status, alcohol consumption, physical activity, dietary supplement, and total calorie intake from diet; model 3 additionally included BMI, diabetes, CVD, cancer, hypertension, and hyperlipidemia.

[b] ORs (95% CIs) of DDS with sarcopenia's components were examined using Logistic regression models; model 1 was adjusted for age and sex; model 2 additionally included race, household income, residence, smoking status, alcohol consumption, physical activity, dietary supplement, total calorie intake from diet, and three components (low muscle strength, low muscle mass, and low physical performance) were mutually adjusted; model 3 additionally included BMI, diabetes, CVD, cancer, hypertension, and hyperlipidemia.

Abbreviations: BMI, body mass index; CIs: confidence intervals; CVD, cardiovascular disease; DDS, dietary diversity score; ORs: odds ratios; Ref, reference; SD, standard deviation.

Table S12 Sensitivity analysis of combined associations of E-DII and DDS with sarcopenia while redefining sarcopenia following the EWGSOP1's recommendations [a]

DDS levels	E-DII (ORs, 95% CIs) [b]			RERI (95% CI) [d]		*P*-interaction [e]
	Tertile 3 [c]	Tertile 2 [c]	Tertile 1 [c]	Tertile 2 of E-DII	Tertile 1 of E-DII	
						< 0.001
Low-level DDS [c]	1.00	0.91 (0.83, 1.00)	0.98 (0.84, 1.13)			
Medium-level DDS [c]	0.84 (0.80, 0.88)	0.77 (0.73, 0.81)	0.76 (0.72, 0.81)	0.05 (-0.04, 0.12)	0.00 (-0.10, 0.09)	
High-level DDS [c]	0.81 (0.75, 0.87)	0.71 (0.67, 0.75)	0.67 (0.63, 0.72)	-0.09 (-0.37, 0.22)	-0.08 (-0.41, 0.17)	

[a] We defined sarcopenia as having low muscle mass and either low muscle strength or low physical performance following the EWGSOP1's recommendations.

[b] Combined associations of E-DII and DDS with sarcopenia were assessed based on covariates in model 3: age, sex, race, household income, residence, smoking status, alcohol consumption, physical activity, BMI, dietary supplement, diabetes, CVD, cancer, hypertension, and hyperlipidemia; three components (low muscle strength, low muscle mass, and low physical performance) were additionally mutually adjusted in

model 3 while assessing the combined associations of E-DII and DDS with sarcopenia' components.

[c] Tertile 1 of E-DII ranged from -5.44 to -0.49, tertile 2 of E-DII ranged from -0.50 to 1.12, and from 1.13 to 4.65 in UK Biobank. DDS categories [low (1-6), medium (7-12), and high (13-18)] were defined according to practical implications for public health.

[d] The estimates of RERI were calculated based on the reference group with tertile 3 of E-DII and low DDS.

[e] Likelihood tests were applied to test the significance of interaction term by comparing the model with and without the interaction term.

Abbreviations: BMI, body mass index; CIs: confidence intervals; CVD, cardiovascular disease; DDS, dietary diversity score; E-DII, dietary inflammatory index; ORs: odds ratios; Ref, reference; RERI, the relative excess risk due to interaction.

Table S13 Sensitivity analysis of combined associations of E-DII and DDS with sarcopenia and its components while excluding participants with extreme BMI [a]

**DDS levels** [b]	**E-DII (ORs, 95% CIs)** [b, c]			**RERI (95% CI)** [d]		***P*-interaction** [e]
	**Tertile 3**	**Tertile 2**	**Tertile 1**	**Tertile 2 of E-DII**	**Tertile 1 of E-DII**	
Sarcopenia						< 0.001
Low-level DDS	1.00	0.53 (0.20, 1.20)	1.19 (0.40, 2.91)			
Medium-level DDS	0.88 (0.62, 1.27)	0.69 (0.48, 1.02)	0.71 (0.48, 1.07)	0.28 (-0.26, 0.82)	-0.36 (-1.55, 0.84)	
High-level DDS	0.80 (0.48, 1.31)	0.63 (0.41, 0.99)	0.60 (0.39, 0.92)	0.30 (-0.31, 0.91)	-0.40 (-1.63, 0.83)	
Low muscle strength						0.871
Low-level DDS	1.00	0.82 (0.67, 1.00)	1.07 (0.80, 1.41)			
Medium-level DDS	0.79 (0.71, 0.87)	0.72 (0.65, 0.81)	0.71 (0.63, 0.80)	0.12 (-0.06, 0.29)	-0.15 (-0.45, 0.16)	
High-level DDS	0.74 (0.64, 0.87)	0.71 (0.63, 0.81)	0.62 (0.55, 0.70)	0.15 (-0.05, 0.35)	-0.20 (-0.52, 0.12)	
Low muscle mass						0.885

Low-level DDS	1.00	1.02 (0.80, 1.28)	0.81 (0.55, 1.18)			
Medium-level DDS	0.92 (0.82, 1.04)	0.88 (0.78, 1.00)	0.75 (0.66, 0.86)	-0.07 (-0.35, 0.21)	0.02 (-0.30, 0.34)	
High-level DDS	0.92 (0.78, 1.08)	0.85 (0.74, 0.97)	0.74 (0.65, 0.85)	-0.09 (-0.36, 0.18)	0.01 (-0.32, 0.35)	
Low physical performance						0.007
Low-level DDS	1.00	0.87 (0.73, 1.03)	0.89 (0.67, 1.15)			
Medium-level DDS	0.79 (0.71, 0.87)	0.64 (0.58, 0.71)	0.64 (0.60, 0.71)	-0.01 (-0.17, 0.16)	-0.04 (-0.28, 0.21)	
High-level DDS	0.70 (0.60, 0.81)	0.59 (0.52, 0.67)	0.54 (0.47, 0.62)	-0.02 (-0.21, 0.17)	0.01 (-0.25, 0.27)	

[a] Extreme BMI was defined as low (<14 kg/m^2) or high (≥36 kg/m^2) BMI.

[b] Tertile 1 of E-DII ranged from -5.45 to -0.50, tertile 2 of E-DII ranged from -0.51 to 1.13, and from 1.14 to 4.65 in UK Biobank. DDS categories [low (1-6), medium (7-12), and high (13-18)] were defined according to practical implications for public health.

[c] Combined associations of E-DII and DDS with sarcopenia were assessed based on covariates in model 3: age, sex, race, household income, residence, smoking status, alcohol consumption, physical activity, dietary supplement, BMI, diabetes, CVD, cancer, hypertension, and

hyperlipidemia; three components (low muscle strength, low muscle mass, and low physical performance) were additionally mutually adjusted in model 3 while assessing the combined associations of E-DII and DDS with sarcopenia' components.

[d] The estimates of RERI were calculated based on the reference group with tertile 3 of E-DII and low DDS.

[e] Likelihood tests were applied to test the significance of interaction term by comparing the model with and without the interaction term.

Abbreviations: BMI, body mass index; CIs: confidence intervals; CVD, cardiovascular disease; DDS, dietary diversity score; E-DII, dietary inflammatory index; ORs: odds ratios; Ref, reference; RERI, the relative excess risk due to interaction.

Table S14 Sensitivity analysis of combined associations of E-DII and DDS with sarcopenia and its components while restricting the analysis to individuals who participated in at least two 24-h dietary surveys (n = 96,133)

DDS levels [a]	E-DII (ORs, 95% CIs) [a, b]			RERI (95% CI) [c]		*P*-interaction [d]
	Tertile 3	Tertile 2	Tertile 1	Tertile 2 of E-DII	Tertile 1 of E-DII	
Sarcopenia						< 0.001
Low-level DDS	1.00 (Ref)	0.89 (0.72, 0.98)	0.75 (0.95, 1.15)			
Medium-level DDS	1.00 (0.61, 1.33)	0.76 (0.72, 0.84)	0.63 (0.49, 0.89)	-0.12 (-0.37, 0.23)	0.03 (-0.30, 0.36)	
High-level DDS	0.86 (0.53, 1.14)	0.67 (0.57, 1.10)	0.59 (0.44, 0.76)	-0.08 (-0.47, 0.52)	-0.03 (-0.47, 0.29)	
Low muscle strength						0.111
Low-level DDS	1.00 (Ref)	1.12 (0.53, 1.65)	1.03 (0.63, 1.52)			
Medium-level DDS	0.79 (0.60, 0.95)	0.72 (0.55, 0.96)	0.69 (0.52, 0.93)	-0.27 (-0.54, 0.40)	0.07 (-0.29, 0.44)	
High-level DDS	0.78 (0.58, 0.97)	0.74 (0.56, 0.99)	0.63 (0.48, 0.85)	-0.16 (-0.45, 0.33)	0.33 (-0.24, 0.70)	
Low muscle mass						0.058

Low-level DDS	1.00 (Ref)	0.93 (0.75, 1.07)	0.70 (0.59, 0.94)			
Medium-level DDS	0.78 (0.57, 0.97)	0.71 (0.52, 0.93)	0.57 (0.42, 0.80)	0.00 (-0.27, 0.33)	0.19 (-0.19, 0.50)	
High-level DDS	0.75 (0.54, 0.95)	0.70 (0.51, 0.91)	0.55 (0.40, 0.79)	0.11 (-0.27, 0.45)	0.15 (-0.23, 0.56)	
Low physical performance						0.024
Low-level DDS	1.00 (Ref)	0.78 (0.45, 1.07)	0.76 (0.42, 1.03)			
Medium-level DDS	0.69 (0.56, 0.86)	0.55 (0.44, 0.68)	0.52 (0.42, 0.66)	0.07 (-0.40, 0.55)	0.07 (-0.88, 1.02)	
High-level DDS	0.58 (0.46, 0.74)	0.53 (0.42, 0.64)	0.47 (0.37, 0.62)	0.11 (-0.37, 0.59)	0.19 (-0.56, 0.84)	

[a] Tertile 1 of E-DII ranged from -5.21 to -0.50, tertile 2 of E-DII ranged from -0.51 to 1.13, and from 1.14 to 4.52 in UK Biobank. DDS categories [low (1-6), medium (7-12), and high (13-18)] were defined according to practical implications for public health.

[b] Combined associations of E-DII and DDS with sarcopenia were assessed based on covariates in model 3: age, sex, race, household income, residence, smoking status, alcohol consumption, physical activity, dietary supplement, BMI, diabetes, CVD, cancer, hypertension, and hyperlipidemia; three components (low muscle strength, low muscle mass, and low physical performance) were additionally mutually adjusted in

model 3 while assessing the combined associations of E-DII and DDS with sarcopenia' components.

[c] The estimates of RERI were calculated based on the reference group with tertile 3 of E-DII and low DDS.

[d] Likelihood tests were applied to test the significance of interaction term by comparing the model with and without the interaction term.

Abbreviations: BMI, body mass index; CIs: confidence intervals; CVD, cardiovascular disease; DDS, dietary diversity score; E-DII, dietary inflammatory index; ORs: odds ratios; Ref, reference; RERI, the relative excess risk due to interaction.

Table S15 Sensitivity analysis of combined associations of E-DII and DDS with sarcopenia and its components when all missing covariates were imputed using multiple imputations (*n* = 202,708)

**DDS levels** [a]	**E-DII (ORs, 95% CIs)** [a, b]			**RERI (95% CI)** [c]		***P*-interaction** [d]
	**Tertile 3**	**Tertile 2**	**Tertile 1**	**Tertile 2 of E-DII**	**Tertile 1 of E-DII**	
Sarcopenia						0.003
Low-level DDS	1.00 (Ref)	0.67 (0.35, 1.19)	0.85 (0.30, 0.92)			
Medium-level DDS	0.95 (0.73, 1.27)	0.84 (0.63, 1.12)	0.80 (0.61, 1.10)	0.21 (-0.25, 0.68)	0.06 (-0.75, 0.87)	
High-level DDS	1.02 (0.68, 1.51)	0.80 (0.57, 1.12)	0.62 (0.40, 0.93)	0.10 (-0.48, 0.68)	-0.06 (-0.93, 0.82)	
Low muscle strength						0.147
Low-level DDS	1.00 (Ref)	0.94 (0.82, 1.08)	0.83 (0.63, 1.07)			
Medium-level DDS	0.76 (0.70, 0.82)	0.73 (0.67, 0.79)	0.71 (0.65, 0.77)	0.12 (-0.01, 0.26)	0.02 (-0.21, 0.24)	
High-level DDS	0.69 (0.61, 0.79)	0.66 (0.59, 0.73)	0.60 (0.54, 0.66)	0.12 (-0.04, 0.28)	-0.03 (-0.26, 0.21)	
Low muscle mass						0.071

Low-level DDS	1.00 (Ref)	0.92 (0.79, 1.07)	0.80 (0.58, 1.06)			
Medium-level DDS	0.89 (0.81, 0.98)	0.89 (0.81, 0.98)	0.87 (0.78, 0.96)	0.08 (-0.07, 0.24)	-0.03 (-0.28, 0.23)	
High-level DDS	0.91 (0.79, 1.05)	0.85 (0.76, 0.95)	0.85 (0.76, 0.95)	0.02 (-0.16, 0.21)	-0.07 (-0.34, 0.21)	
Low physical performance						<0.001
Low-level DDS	1.00 (Ref)	0.90 (0.79, 1.01)	0.88 (0.72, 1.06)			
Medium-level DDS	0.73 (0.69, 0.79)	0.64 (0.60, 0.69)	0.62 (0.57, 0.66)	-0.03 (-0.15, 0.08)	0.00 (-0.18, 0.18)	
High-level DDS	0.60 (0.53, 0.67)	0.56 (0.52, 0.60)	0.53 (0.49, 0.58)	-0.04 (-0.17, 0.10)	0.05 (-0.13, 0.24)	

[a] Tertile 1 of E-DII ranged from -5.44 to -0.46, tertile 2 of E-DII ranged from -0.47 to 1.17, and from 1.18 to 4.72 in UK Biobank. DDS categories [low (1-6), medium (7-12), and high (13-18)] were defined according to practical implications for public health.

[b] Combined associations of E-DII and DDS with sarcopenia were assessed based on covariates in model 3: age, sex, race, household income, residence, smoking status, alcohol consumption, physical activity, dietary supplement, BMI, diabetes, CVD, cancer, hypertension, and hyperlipidemia; three components (low muscle strength, low muscle mass, and low physical performance) were additionally mutually adjusted in

model 3 while assessing the combined associations of E-DII and DDS with sarcopenia' components.

[c] The estimates of RERI were calculated based on the reference group with tertile 3 of E-DII and low DDS.

[d] Likelihood tests were applied to test the significance of interaction term by comparing the model with and without the interaction term.

Abbreviations: BMI, body mass index; CIs: confidence intervals; CVD, cardiovascular disease; DDS, dietary diversity score; E-DII, dietary inflammatory index; ORs: odds ratios; Ref, reference; RERI, the relative excess risk due to interaction.

Table S16 Subgroup analyses of associations of E-DII with sarcopenia and its components [a]

Subgroups	Sarcopenia	Low muscle strength	Low muscle mass	Low physical performance
Age groups				
≥55 years	1.13 (0.96, 1.32)	1.06 (1.02, 1.10)	1.05 (1.02, 1.08)	1.09 (1.06, 1.13)
<55 years	1.09 (1.01, 1.17)	1.04 (1.02, 1.07)	1.08 (1.05, 1.10)	1.08 (1.06, 1.11)
P-interaction [b]	0.284	0.330	0.640	0.388
Sex				
Females	1.02 (1.03, 1.21)	1.06 (1.04, 1.09)	1.07 (1.05, 1.09)	1.09 (1.07, 1.12)
Males	1.04 (0.93, 1.16)	1.05 (1.02, 1.08)	1.09 (1.04, 1.14)	1.09 (1.06, 1.12)
P-interaction [c]	0.078	0.274	0.438	0.887
BMI groups				
≥25 kg/m^2	0.83 (0.42, 1.62)	1.05 (1.03, 1.08)	1.18 (1.02, 1.36)	1.08 (1.06, 1.10)
<25 kg/m^2	1.09 (1.02, 1.16)	1.06 (1.03, 1.10)	1.03 (1.01, 1.04)	1.16 (1.11, 1.21)

P-interaction [d]	0.384	0.351	0.081	0.052

[a] Associations of E-DII with sarcopenia were assessed based on covariates in model 3: age, sex, race, household income, residence, smoking status, alcohol consumption, physical activity, BMI, dietary supplement, diabetes, CVD, cancer, hypertension, and hyperlipidemia; three components (low muscle strength, low muscle mass, and low physical performance) were additionally mutually adjusted in model 3 while assessing the associations of E-DII with sarcopenia' components.

[b] The interactions were tested by comparing the -2 log-likelihood values in the models with and without the cross-product interaction term of age and E-DII.

[c] The interactions were tested by comparing the -2 log-likelihood values in the models with and without the cross-product interaction term of sex and E-DII.

[d] The interactions were tested by comparing the -2 log-likelihood values in the models with and without the cross-product interaction term of BMI and E-DII.

Abbreviations: BMI, body mass index; CIs: confidence intervals; CVD, cardiovascular disease; E-DII, dietary inflammatory index; ORs: odds ratios.

Table S17 Subgroup analyses of associations of DDS with sarcopenia and its components [a]

Subgroups	Sarcopenia	Low muscle strength	Low muscle mass	Low physical performance
Age groups				
≥55 years	1.04 (0.96, 1.12)	0.98 (0.96, 1.00)	1.00 (0.99, 1.02)	0.97 (0.95, 0.98)
<55 years	0.96 (0.93, 0.99)	0.97 (0.96, 0.98)	0.99 (0.97, 1.00)	0.94 (0.93, 0.95)
P-interaction [b]	0.268	0.421	0.062	0.087
Sex				
Females	0.98 (0.94, 1.02)	0.97 (0.95, 0.98)	0.99 (0.98, 1.00)	0.95 (0.94, 0.96)
Males	0.94 (0.89, 1.00)	0.96 (0.95, 0.98)	0.98 (0.96, 1.00)	0.95 (0.94, 0.96)
P-interaction [c]	0.293	0.406	0.173	0.343
BMI groups				
≥25 kg/m^2	1.01 (0.72, 1.43)	0.97 (0.96, 0.98)	1.00 (0.93, 1.07)	0.95 (0.94, 0.96)
<25 kg/m^2	0.97 (0.94, 1.00)	0.96 (0.94, 0.97)	1.00 (0.99, 1.01)	0.92 (0.90, 0.94)

P-interaction [d]	0.737	0.803	0.670	0.057

[a] Associations of DDS with sarcopenia were assessed based on covariates in model 3: age, sex, race, household income, residence, smoking status, alcohol consumption, physical activity, BMI, total calorie intake from diet, dietary supplement, diabetes, CVD, cancer, hypertension, and hyperlipidemia; three components (low muscle strength, low muscle mass, and low physical performance) were additionally mutually adjusted in model 3 while assessing the associations of DDS with sarcopenia' components.

[b] The interactions were tested by comparing the -2 log-likelihood values in the models with and without the cross-product interaction term of age and DDS.

[c] The interactions were tested by comparing the -2 log-likelihood values in the models with and without the cross-product interaction term of sex and DDS.

[d] The interactions were tested by comparing the -2 log-likelihood values in the models with and without the cross-product interaction term of BMI and DDS.

Abbreviations: BMI, body mass index; CIs: confidence intervals; CVD, cardiovascular disease; DDS, dietary diversity score; ORs: odds ratios.

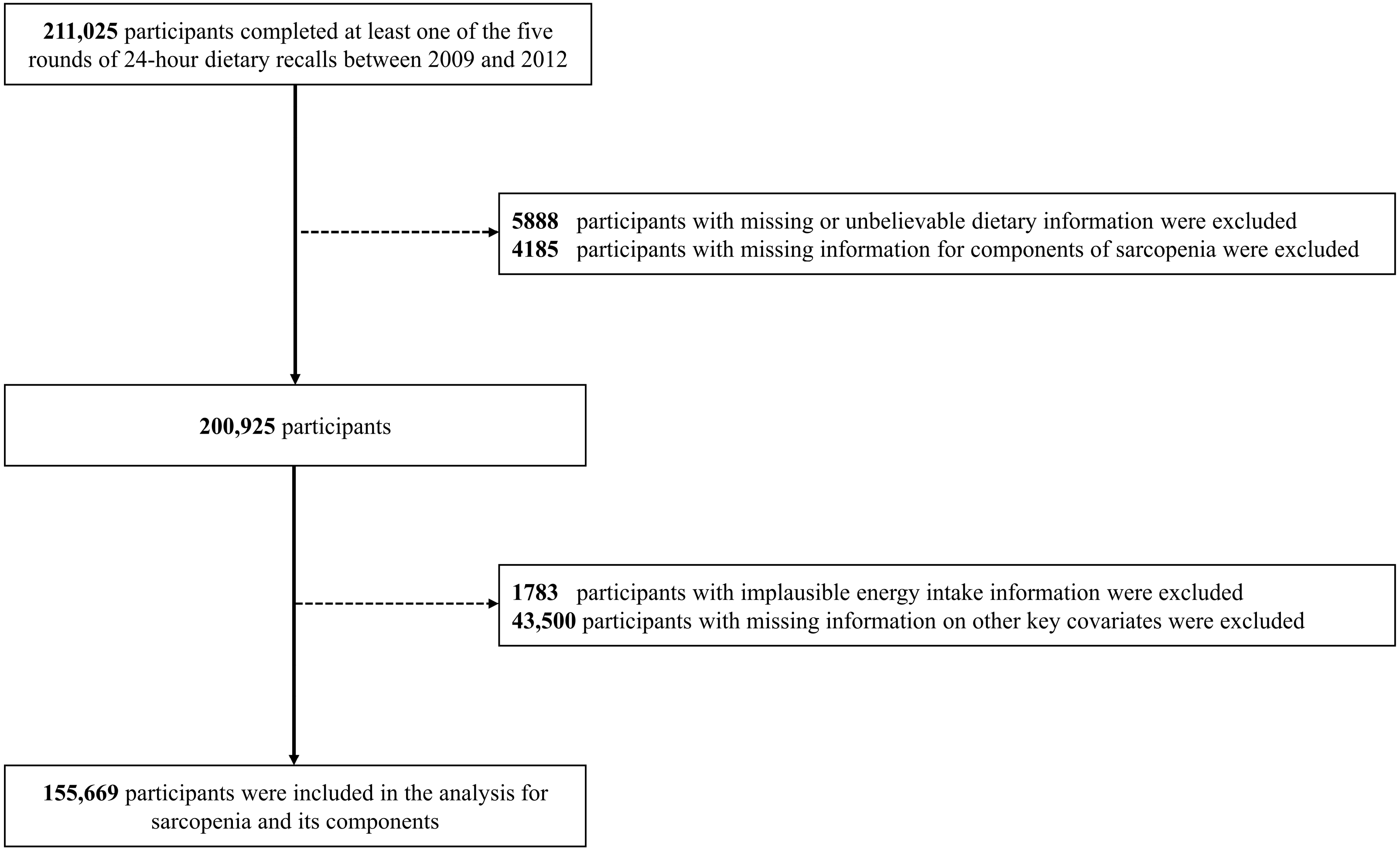

Figure S1 Flow chart for UK Biobank using 24-h dietary assessment data**.**

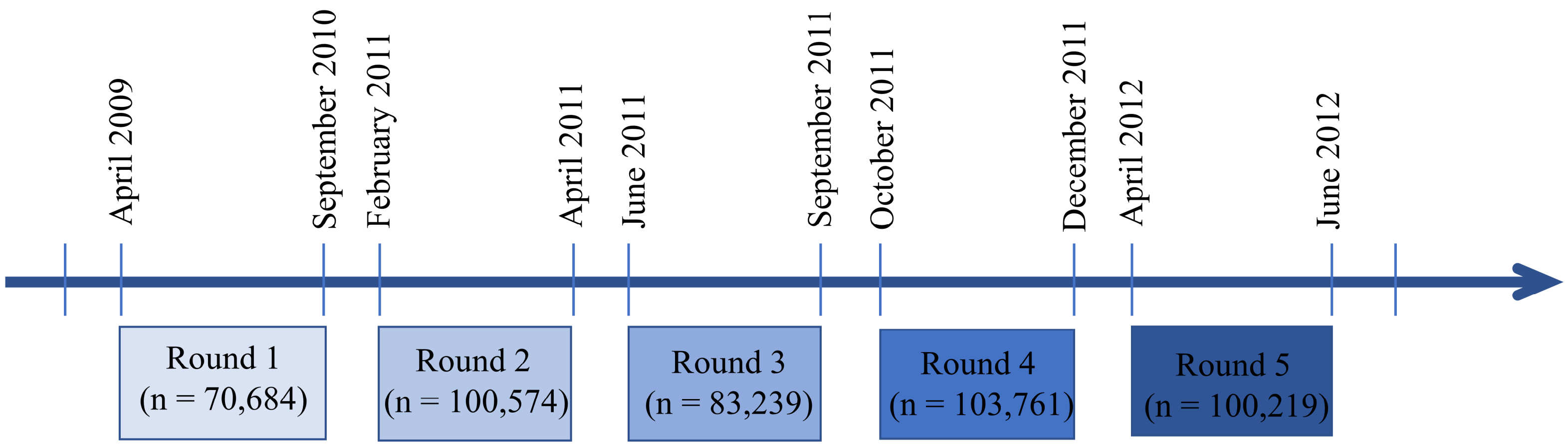

Figure S2 Timeline of five rounds of 24-hour dietary recall surveys in UK Biobank.

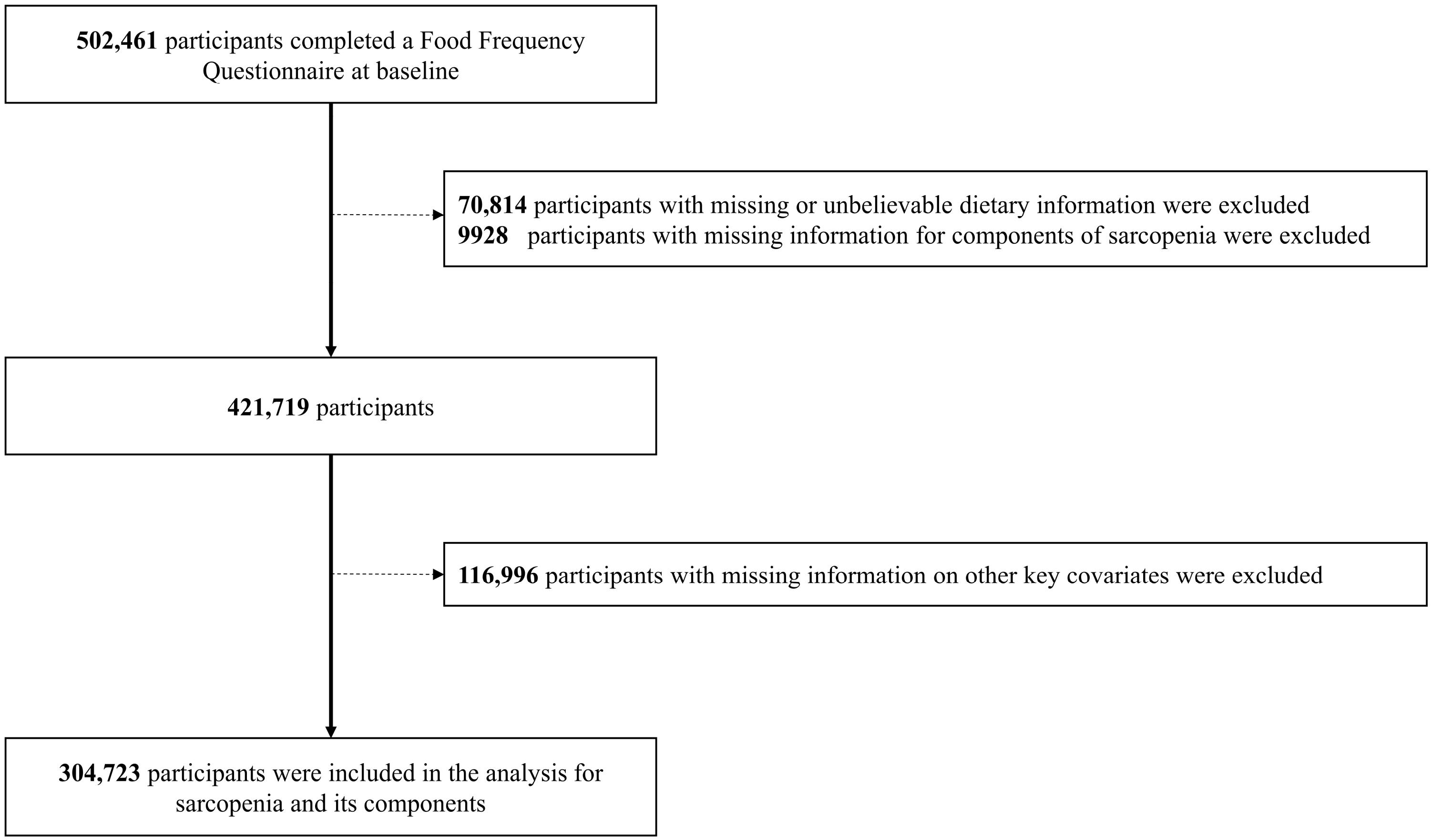

Figure S3 Flow chart for UK Biobank using Food Frequency Questionnaire data**.**

Reference

1. Dodds, R.M.; Granic, A.; Robinson, S.M.; Sayer, A.A. Sarcopenia, long-term conditions, and multimorbidity: findings from UK Biobank participants. *J Cachexia Sarcopenia Muscle* **2020**, *11*, 62-68, doi:10.1002/jcsm.12503.
2. Petermann-Rocha, F.; Ferguson, L.D.; Gray, S.R.; Rodriguez-Gomez, I.; Sattar, N.; Siebert, S.; Ho, F.K.; Pell, J.P.; Celis-Morales, C. Association of sarcopenia with incident osteoporosis: a prospective study of 168,682 UK biobank participants. *J Cachexia Sarcopenia Muscle* **2021**, *12*, 1179-1188, doi:10.1002/jcsm.12757.
3. WHO. International guide for monitoring alcohol consumption and related harm. **2000**, 51-52.
4. Bassett, D.R., Jr. International physical activity questionnaire: 12-country reliability and validity. *Med Sci Sports Exerc* **2003**, *35*, 1396, doi:10.1249/01.MSS.0000078923.96621.1D.
5. Madjedi, K.M.; Stuart, K.V.; Chua, S.Y.L.; Ramulu, P.Y.; Warwick, A.; Luben, R.N.; Sun, Z.; Chia, M.A.; Aschard, H.; Wiggs, J.L., et al. The Association of Physical Activity with Glaucoma and Related Traits in the UK Biobank. *Ophthalmology* **2023**, *130*, 1024-1036, doi:10.1016/j.ophtha.2023.06.009.
6. Traversy, G.; Chaput, J.-P. Alcohol Consumption and Obesity: An Update. *Current Obesity Reports* **2015**, *4*, 122-130, doi:10.1007/s13679-014-0129-4.
